# Impact of the ventral hernia working group’s publication: a bibliometric analysis

**DOI:** 10.1007/s10029-024-03093-x

**Published:** 2024-06-18

**Authors:** Sara M. Maskal, Sergio Mazzola Poli de Figueiredo, Matthew Weaver, Mary Schleicher, Chao Tu, Ryan C. Ellis, Kimberly Woo, Aldo Fafaj, Daphne Remulla, Benjamin T. Miller, Clayton C. Petro, Lucas R.A. Beffa, Ajita S. Prabhu, Michael J. Rosen

**Affiliations:** 1 Department of Surgery, Cleveland Clinic, 2049 E 100th St, Desk A-100, Cleveland, OH 44106 USA; 2 Floyd D. Loop Alumni Library, Cleveland Clinic, Cleveland, OH USA

**Keywords:** Ventral hernia repair, Comorbidity, Bibliometric analysis, Hernia mesh, Ventral hernia Working Group

## Abstract

**Purpose:**

The Ventral Hernia Working Group (VHWG) proposed a ventral hernia grading guideline, primarily supported by expert opinion, recommending biologic mesh placement in high-risk patients. We investigated the relationship between this industry-sponsored guideline and discourse around ventral hernia repair (VHR).

**Methods:**

Medline platform from Web of Science’s database identified publications “pre-VHWG”(1999-01-01 to 2009-12-31), and “post-VHWG”(2010-01-01 to 2020-12-31) describing VHR and complications or recurrence of VHR with the following comorbidities: COPD, smoking, diabetes, immunosuppression, or obesity. Poisson regression analyzed keyword frequency over time using logarithmically transformed data.

**Results:**

Of 1291 VHR publications identified pre-VHWG and 3041 publications identified post-VHWG, 172 (13.3%) and 642 (21.1%) publications respectively included prespecified keywords. The keyword groups “biologic”(IRR 3.39,95%CI1.34-11.4,*p* = 0.022) and “comorbid”(IRR 1.95, 95%CI1.09-3.74,p = 0.033) significantly increased with frequency after publication of the VHWG.

**Conclusion:**

The VHWG publication likely contributed to a focus on comorbidities and biologic mesh in the ensuing literature within the field of VHR.

**Supplementary Information:**

The online version contains supplementary material available at 10.1007/s10029-024-03093-x.

## Introduction

It is well established that medical drug and device industries can affect national research agendas through subtle influences at various levels across the healthcare marketplace [1–3]. These include evidence base production, evidence synthesis, understanding of harms issues, cost-effectiveness evaluation, clinical guidelines formation, healthcare professional education and direct influences on healthcare professional decisions [3]. Although various relationships between mesh companies and hernia practice and outcomes have been explored [4–6], a knowledge gap persists regarding whether industry relationships have affected the broader hernia research agenda with respect to the types of research questions that are studied or the assumptions on which most studies are based. It is possible to investigate the effect of certain publications on the future scientific evaluation of key terms utilized in these sponsored publications by utilizing bibliometric analysis. Popular uses for bibliometric analyses include the identification of topical trends and identification of the most influential authors and papers within a field [7–9].

In 2010, the Ventral Hernia Working Group (VHWG) published a classification system to grade incisional hernias based on patient risk factors for wound complications which was funded by LifeCell (LifeCell Corp, Branchburg, NJ, USA), a company that markets biologic mesh [10]. In acknowledgement of a lack of high quality data and existing grading system for evaluating patient risk factors in ventral hernia repair, the group developed a 4-level grading scheme that was largely based on the consensus expert opinions of the authors. Grading comprised patient comorbidities (smoking, obesity, diabetes, immunosuppression, COPD), history of prior wound infection, and the level of contamination involved in the case. The VHWG provided recommendations for mesh selection based on these Hernia Grades, including a suggestion that biologic materials harbored potential advantages in Grade 2–4 hernias. Notably, at the time of publication there were no randomized trials comparing synthetic and biologic mesh use in ventral hernia repair. Subsequent authors have investigated the validity of the VHWG’s assertions that certain patient characteristics presented prohibitive risks of wound complications if permanent synthetic mesh was used for reinforcement of ventral hernia repairs with variable results [11–15]. While it is known that the publication was widely cited, to our knowledge there has been no investigation of how the publication changed the frequency of specific topics written about in the field [8]. The impact of the VHWG publication on subsequent literature with particular interest in the discussion around comorbidities’ effect on outcomes in ventral hernia repair and the utilization of biologic mesh in hernia repairs is unknown. Utilizing bibliometric analysis, we aimed to evaluate the impact of the 2010 VHWG publication on the subsequent literature on ventral hernia repair. Specifically, we sought to quantify the effect of the publication on prevalence of the topics highlighted by VHWG in successive manuscripts within the field of ventral hernia repair.

## Methods

This study was exempt from Institutional Review Board/ethical committee review and did not require consent as there were no human subjects involved. The search strategy below was created to capture the literature describing incisional or ventral hernia repair in patients as well as complications or recurrence of incisional or ventral hernia repair in patients with any of the following five comorbidities: COPD, smoking, diabetes, immunosuppression, or obesity. The Medline platform from Clarivate’s Web of Science database was searched on October 27, 2023. A combination of keywords using the “Topic” (TS) field as well as medical subject headings (MHX) were used where appropriate to identify key topics within the literature (Supplement 1). The key topics relevant to the VHWG were agreed upon by co-investigators and were as follows: (1) chronic obstructive pulmonary disease (COPD), (2) smoking (3) diabetes, (4) immunosuppression, (5) obesity, (6) comorbidity, and (7) biologic. To identify and analyze the impact of the 2010 paper by the Ventral Hernia Working Group, the search was divided into two periods: “pre-VHWG” (1999-01-01 to 2009-12-31), and “post-VHWG” (2010-01-01 to 2020-12-31). Relevance of search topics was manually reviewed for consensus by two co-investigators and discrepancies were resolved by consensus. Articles were included in the denominator of publications in the field if the abstract explicitly mentioned and was focused on ventral hernia repair. Articles were included for the key topic group if they mentioned the previously listed key topics relevant to the VHWG in the context of ventral hernias. Articles that did not mention or were unrelated to ventral hernia repairs were excluded.

### Statistical analysis

Citation analysis was performed using Web of Science Expanded API Expanded (https://developer.clarivate.com/apis/wos) with a custom script written in the Python programming language version 3.11.5 (https://www.python.org/). Author citation reports were downloaded from the Web of Science website and the Disruption Index of the Ventral Hernia Working Group publication was calculated according to Wu et al. [16] The Disruption Index is a score calculated based on the ratio of future papers citing the focal paper alone compared to citations of the focal paper’s references. The score ranges from − 1 to 1, with negative values indicating “developmental” papers that reinforce current thinking and positive numbers indicating “disruptive” papers, which introduce new ideas [16]. Poisson regression analysis was used analyze keyword count data over time modeled using the logarithmically transformed publication data. In the regression model, we included the indicator of topics mentioned before or after 2010, the name of fields and years. Common effect test, average relative test and heterogeneity tests were performed on this data to identify the most appropriate model. All statistical analysis was completed using R software package version 4.3.2 (The R Foundation, Vienna, Austria).

## Results

There were 1291 publications on the topic of incisional hernia repairs identified in the decade pre-VHWG and 3041 publications identified in the decade post-VHWG. Of those publications on incisional hernia repairs, pre-VHWG there were 172 publications and 642 publications post-VHWG that included one of the prespecified key topics from the VHWG.

**Fig. 1:**
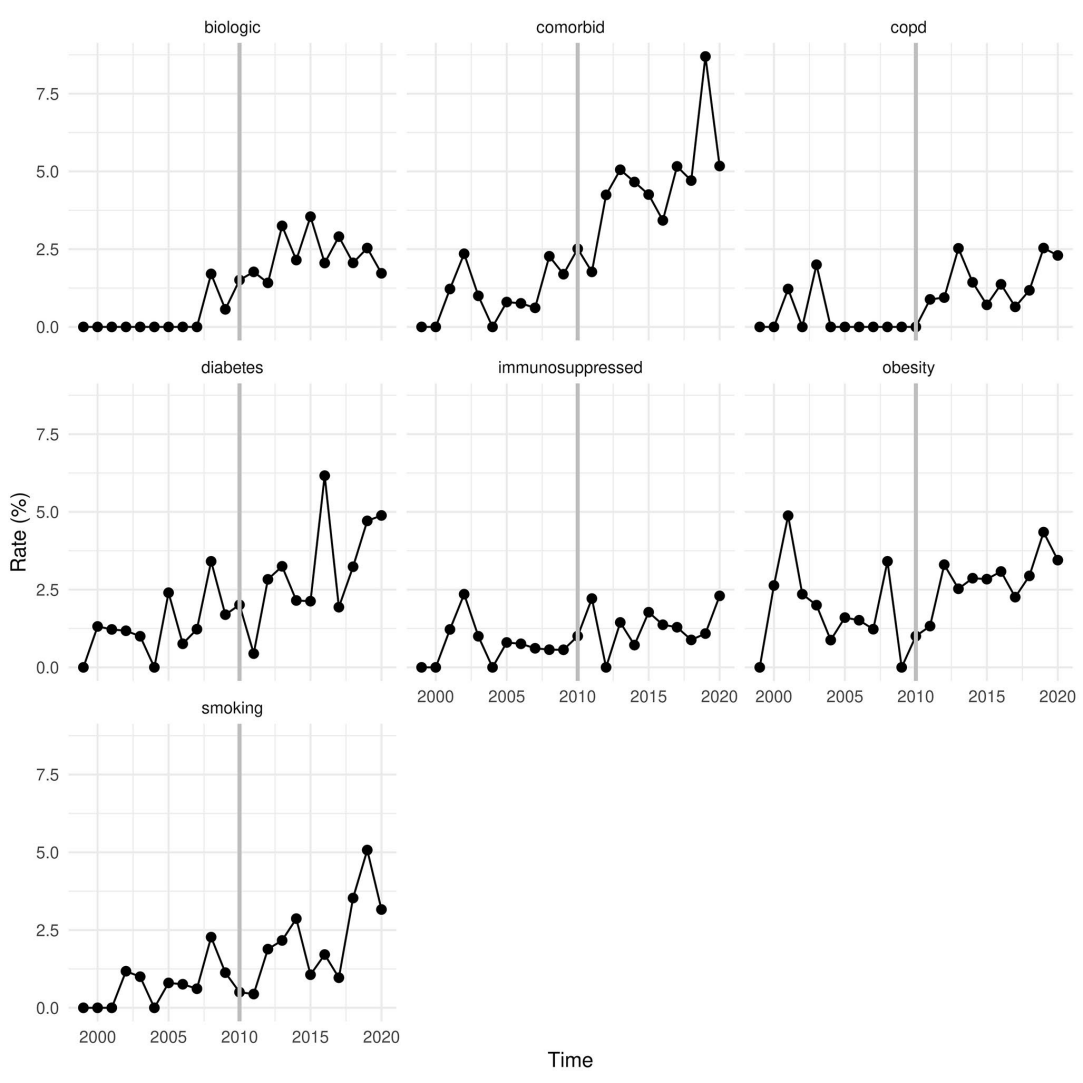
Rates of topic frequency over time

The prespecified topics generally increased with frequency over the study period (Fig. 1). On further analysis, there was not a common effect between topics (RR 1.27, *p* = 0.20) or average relative effect (RR 1.44, *p* = 0.071), but the dataset was heterogeneous (*p* = 0.097). Individually, the topics “biologic” (IRR 3.39, 95%CI 1.34–11.4, *p* = 0.022) and “comorbid” (IRR 1.95, 95% 1.09–3.74, *p* = 0.033) were the only topics that significantly increased with frequency after publication of the VHWG (Table 1).

**Table 1:** Incidence of keywords pre-VHWG and post-VHWG

Characteristic	IRR^1^	95% CI^1^	*p*-value
Keyword groups pre-VHWG			
Biologic	0.00	0.00, 0.00	< 0.001
Comorbid	0.00	0.00, 0.00	< 0.001
COPD	0.00	0.00, 0.00	< 0.001
Diabetes	0.00	0.00, 0.00	< 0.001
Immunosuppressed	0.00	0.00, 0.00	< 0.001
Obesity	0.00	0.00, 0.00	< 0.001
Smoking	0.00	0.00, 0.00	< 0.001
Year	1.08	1.05, 1.11	< 0.001
Keyword groups post-VHWG			
After 2010 * Biologic	3.39	1.34, 11.4	0.022
After 2010 * Comorbid	1.95	1.09, 3.74	0.033
After 2010 * COPD	2.71	0.95, 11.5	0.10
After 2010 * Diabetes	0.99	0.57, 1.78	> 0.9
After 2010 * Immunosuppressed	0.86	0.41, 1.98	0.7
After 2010 * Obesity	0.72	0.42, 1.25	0.2
After 2010 * Smoking	1.20	0.62, 2.51	0.6

^1^IRR = Incidence Rate Ratio, CI = Confidence Interval

*Certain data included herein are derived from the ? Web of Science (Jan 29, 2024) of Clarivate. All rights reserved. You may not copy or re-distribute this material in whole or in part without the prior written consent of Clarivate.

After VHWG publication, there were 195 publications citing only the index paper (VHWG), 505 publications citing the index and at least one of the references from the index paper, and no papers citing the references without citing VHWG. The calculated disruption index for the VHWG publication was − 0.44.

## Discussion

The decade after publication of the Ventral Hernia Working Group in 2010 demonstrated an increased frequency of publications focused on biologic mesh and comorbidities compared to the preceding decade. Based on a disruption index of -0.44, the Ventral Hernia Working Group publication reinforced concepts existing in the literature of the time regarding the importance of comorbidities and the role of biologic mesh in VHR.

Bibliometric analyses have been used in multiple medical fields to synthesize large amounts of data and identify publication trends and the impact of authors, publications institutions, and journals within their respective fields. For example, these methods have been used to demonstrate the evolution of keywords related to Enhanced Recovery After Surgery (ERAS) protocols and the most influential authors, institutions and journals in the field over a period of 20 years [17]. Similar analyses have been performed on the topic of inguinal and ventral hernias to demonstrate the most influential authors and journals within the fields[7–9, 18]. One such analysis recently identified the Ventral Hernia Working Group publication as the second most frequently cited paper on the topic of incisional hernias and found that “acellular dermal matrix” was one of the top 20 keyw ords with the strongest citation bursts after 2010[8]. To our knowledge, the application of these methods to identify the impact of a single publication linked to industry sponsors on the discourse within a field is relatively novel.

The primary aim of this work was to evaluate the impact of the VHWG publication on the trend of topics in subsequent literature, which was assessed through keyword analysis. We found a significant increase in the frequency of use of the terms “comorbid” and “biologic” in publications on incisional hernia repair after 2010. The Disruption Index (DI) and the high number of citations demonstrated the broader impact of the VHWG publication on the field. The DI for the VHWG publications was considered “developmental”, which suggests that it reviewed and potentially propagated themes that were already existing in the field. Notably, this does not provide a critique on the validity of those existing themes and fails to fully capture that some concepts in the paper may be novel. For example, it might be fair to surmise that the VHWG was reinforcing existing ideas that obesity, immunosuppression, and smoking are problematic in hernia repair, however the idea that biologic mesh is beneficial for high-risk, comorbid patients was relatively avant-garde.

The topic of patient comorbidities as they relate to ventral hernia repair outcomes increased in prevalence in the literature following the VHWG publication. This persists today as evidenced by the 2023 European Hernia Society guidelines recommending preoptimization for patients with obesity, diabetes, and smoking [19] as well as many hernia surgeons’ adoption of hard cut-offs for preoperative optimization. However, the concept of preoptimization in the setting of ventral hernia repair is multifaceted and controversial [20]. While an evaluation of the evidence for or against preoptimization of patient comorbidities is beyond the scope of this work, our data suggests that the VHWG publication may have increased the focus on this topic. Ultimately, most of the data on these topics (i.e. smoking, obesity, diabetes, etc.) is retrospective, which carries inherent limitations and leaves room for debate [12–15, 21–23]. Our data suggests that the VHWG publication may have encouraged surgeons to focus on comorbidities in hernia repair; a narrative that has persisted.

The VWHG grading system recommended the use of biologic mesh to offset risk operative risk associated with comorbidities. Our work suggests that this likely promoted the frequency of discussion about biologic mesh in the literature. Acellular dermal matrix has been used for burn wounds since the 1990s and for ventral hernia repair since the early 2000s [24–26]. Our data suggests that biologics became more frequently discussed in ventral hernia literature after this publication in 2010. The sponsor of the publication is a producer of biologic mesh. While the effect of financial sponsorship on specific publications is outside of the scope of the current work, it may be an interesting avenue for future investigation.

Our study has several limitations that should be addressed. We limited the search to the WoS database to improve accuracy for document type and obtain citation report function in contrast to Scopus and Pubmed [8, 27, 28]. The co-investigators set the search strategy because the MeSH terms in the VHWG publication were generic, which may present a source of selection bias. Also, as this is a retrospective study, we can describe association but not infer causation. For example, we utilized the VHWG in isolation and could not account for other influential literature or factors that could have played a role in the trends noted by our study. For example, key opinion leaders in the field likely contributed to the usage through other publications and speakerships. The increased interest in biologic mesh may also have reflected the tendency of surgeons to investigate novel devices. As in all retrospective work, it is impossible to control for all the potential confounding variables.

## Conclusion

The VHWG publication may have played a part in propagating the use of biologic mesh and a focus on patient comorbidities in ventral hernia repair. While surgeons should be aware of financial conflicts, more scrutiny of evidence levels and bias may be warranted prior to publication of clinical guidelines with industrial sponsors.

## Electronic supplementary material

Below is the link to the electronic supplementary material.

Supplementary Material 1

Supplementary Material 2
