# Supplementary material for: Impact of the ventral hernia working group’s publication: a bibliometric analysis
Source: Hernia. 2024 Jun 18;28(5):1843–8. doi: 10.1007/s10029-024-03093-x (PMC11450067; doi:10.1007/s10029-024-03093-x)
Supplement: Supplementary file 1 — Supplementary Material 1 [file 10029_2024_3093_MOESM1_ESM.docx]

Supplement 1: Search strategy

1     TS= ("incisional hernia*" or" ventral hernia*" or "abdominal wall") NEAR/3 (repair* or reconstruct*)

2     MHX=(Surgical Wound)

3     TS=(wound or wounds or infection* or complication* or recur* or occurrence or seroma* or hematoma*)

4     #2 OR #3

5     MHX= (Pulmonary Disease, Chronic Obstructive OR Smoking OR Diabetes Mellitus OR Comorbidity OR **Glycated Hemoglobin OR Glycemic Control)**

6     TS=( "weight loss" or "body mass index" or BMI or immunosuppress* or "chronic obstructive pulmonary disease" or COPD or obesity or comorbid* or tobacco or optimization or **"glycated hemoglobin" or A1c OR "Hb A1c" or “glucose control” or “glycemic control” or diabetes or diabetic* )**

7     #5 OR #6

8     #1 AND #4 AND #7 = 1,185

9     #8 limited to Timespan: 1999-01-01 to 2009-12-31 (Publication Date)

10   #8 limited to Timespan: 2010-01-01 to 2020-12-31 (Publication Date)
