# Supplementary material for: Impact of the ventral hernia working group’s publication: a bibliometric analysis
Source: Hernia. 2024 Jun 18;28(5):1843–8. doi: 10.1007/s10029-024-03093-x (PMC11450067; doi:10.1007/s10029-024-03093-x)
Supplement: Supplementary file 2 — Supplementary Material 2 [file 10029_2024_3093_MOESM2_ESM.docx]

**Statements and Declarations:**

Funding: This trial received no funding.

Competing Interests: Clayton Petro serves as an Advanced Medical Solutions, Bard-Davol, and Surgimatix Consultant, and has received research grants from the American Hernia Society, the Central Surgical Association, and the Society of American Gastrointestinal and Endoscopic Surgeons. Michael Rosen is the medical director of Abdominal Core Health Quality Collaborative, has a research grant from TelaBio, and has stock options with Ariste. Ajita Prabhu is on the advisory board for Surgimatix, and CMR Surgical. Sara Maskal accepted a resident research grant from the Abdominal Core Health Quality Collaborative. Benjamin Miller has a research grant from Integra. Ryan Ellis, Sergio Mazzola Poli de Figueiredo, Matthew Weaver, Mary Schleicher, Lucas Beffa, Kimberly Woo, Daphne Remulla, Aldo Fafaj, and Chao Tu, have no financial disclosures.
